# Treatment of Human Glioblastoma U251 Cells with Sulforaphane and a Peptide Nucleic Acid (PNA) Targeting miR-15b-5p: Synergistic Effects on Induction of Apoptosis

**DOI:** 10.3390/molecules27041299

**Published:** 2022-02-15

**Authors:** Jessica Gasparello, Chiara Papi, Matteo Zurlo, Laura Gambari, Andrea Rozzi, Alex Manicardi, Roberto Corradini, Roberto Gambari, Alessia Finotti

**Affiliations:** 1 Department of Life Sciences and Biotechnology, University of Ferrara, 44121 Ferrara, Italy; jessica.gasparello@unife.it (J.G.); chiara.papi@unife.it (C.P.); matteo.zurlo@unife.it (M.Z.); 2 Laboratorio RAMSES, IRCCS Istituto Ortopedico Rizzoli, 40136 Bologna, Italy; laura.gambari@ior.it; 3 Department of Chemistry, Life Sciences and Environmental Sustainability, University of Parma, 43124 Parma, Italy; andrea.rozzi@unipr.it (A.R.); alex.manicardi@unipr.it (A.M.); roberto.corradini@unipr.it (R.C.)

**Keywords:** peptide nucleic acids, sulforaphane, glioblastoma, microRNAs, miR-15b-5p, miRNA targeting, combined therapy

## Abstract

Glioblastoma multiforme (GBM) is a lethal malignant tumor accounting for 42% of the tumors of the central nervous system, the median survival being 15 months. At present, no curative treatment is available for GBM and new drugs and therapeutic protocols are urgently needed. In this context, combined therapy appears to be a very interesting approach. The isothiocyanate sulforaphane (SFN) has been previously shown to induce apoptosis and inhibit the growth and invasion of GBM cells. On the other hand, the microRNA miR-15b is involved in invasiveness and proliferation in GBM and its inhibition is associated with the induction of apoptosis. On the basis of these observations, the objective of the present study was to determine whether a combined treatment using SFN and a peptide nucleic acid interfering with miR-15b-5p (PNA-a15b) might be proposed for increasing the pro-apoptotic effects of the single agents. To verify this hypothesis, we have treated GMB U251 cells with SFN alone, PNA-a15b alone or their combination. The cell viability, apoptosis and combination index were, respectively, analyzed by calcein staining, annexin-V and caspase-3/7 assays, and RT-qPCR for genes involved in apoptosis. The efficacy of the PNA-a15b determined the miR-15b-5p content analyzed by RT-qPCR. The results obtained indicate that SFN and PNA-a15b synergistically act in inducing the apoptosis of U251 cells. Therefore, the PNA-a15b might be proposed in a “combo-therapy” associated with SFN. Overall, this study suggests the feasibility of using combined treatments based on PNAs targeting miRNA involved in GBM and nutraceuticals able to stimulate apoptosis.

## 1. Introduction

Glioblastoma multiforme (GBM) is a lethal malignant tumor accounting for 42% of the tumors of the central nervous system, the median survival being 15 months [1,2,3]. At present, no curative treatment is available for GBM and the most used first-line drug, temozolomide (TMZ), is only able to cause an increase in the life expectancy of the treated patients, though this is still not satisfactory [4]. Therefore, new drugs are urgently needed for determining their possible employment in therapeutic protocols for anti-GBM treatments, also tackling important issues of the GBM management, such as the development of drug resistance [5,6,7,8,9,10].

In this context, combined therapy appears to be a very interesting approach for gliomas and other tumors [11,12,13,14,15]. In the case of combined treatments using drugs and/or biomolecules with expected different mechanism(s) of action, it is possible to obtain the same or an increased biological activity with therapeutic potential using sub-optimal concentrations of the single drugs. Furthermore, side effects might be significantly lower when combined treatments are employed [11]. Another very interesting possibility is that combined treatments might allow us to limit the issue of drug resistance, which is an impacting feature of several cancers [11,12,15]. In this respect, a high proportion of GBMs become TMZ-resistant with time [7,8,9,10].

Considering the issue of personalized anti-cancer treatments and patient-to-patient variability in response to drug treatment, combinations of drugs characterized by independent actions might be effective in clinical populations, even in the case of a lack of synergism or additive effects [16]. In fact, as discussed by Palmer et al. [16], each patient will, in this case, benefit solely from the drug to which his/her tumor is highly sensitive, with no clinical improvement from the use of the other drug used in combined protocols [16]. In any case, an increased interest does exist in the development of anti-cancer therapeutic protocols based on combined treatments [12].

We have recently demonstrated that the treatment of glioblastoma cell lines with peptide nucleic acids (PNAs) targeting anti-apoptotic microRNAs (miRNAs) leads to the induction of the apoptosis of glioblastoma cell lines, such as U251 and T98G cell lines. Peptide nucleic acids, first described by Nielsen et al. [17], are DNA analogues in which the sugar–phosphate backbone has been replaced by N-(2-aminoethyl)-glycine units [18]. Despite this important structural change with respect to DNA and RNA, PNAs are capable of sequence-specific and efficient hybridization, forming Watson–Crick double helices with complementary DNA and RNA [17]. For this reason, PNAs have been considered in several biomedical applications, including those that aim to modify gene expression, including an alteration of the biological activity of microRNAs. For instance, we have demonstrated, in a first study, that a peptide nucleic acid targeting miR-221-3p (PNA-a221) induces apoptosis in human glioblastoma U251 cells [19]. In further studies, PNA-a221 was co-delivered together with temozolomide (TMZ) [20], PNA-a222 (targeting miR-222-3p) [21] or PNA-a155 (targeting miR-155-5p) [22]. In all cases, increased effects on apoptosis were found with combined treatments [22].

The rationale for targeting miRNAs is that these short non-coding RNA molecules act as gene regulators, repressing translation or inducing the cleavage of target RNA transcripts [23,24,25]. Emerging evidence suggests that the altered expression of miRNAs may be involved in the pathogenesis of cancer, including GBM [26,27]. Concerning miRNAs involved in glioblastoma, miR-15b-5p has been found up-regulated in aggressive gliomas and its expression correlates with poor prognosis for glioblastoma [26,27,28]. This observation confirmed a previous study published by Xiao et al. [29] who found, after univariate and multivariate Cox regression analysis, two miRNAs (hsa-miR-10b-5p and hsa-miR-15b-5p) that were predictive of high-risk for poor prognosis in glioblastoma patients [29,30]. The involvement of miR-15b-5p in other types of cancers has been also reported [31,32,33,34].

With respect to combined treatments, sulforaphane (SFN) might be of great interest for glioblastomas [35,36,37]. SFN is well established as one of the most important bioactive components of broccoli (*Brassica oleracea* var. *Italica*) and other cruciferous vegetables [38,39,40,41]. SFN is an isothiocyanate generated by the hydrolysis of glucosinolate glucoraphanin by myrosinase (this enzyme is present in plants and gastrointestinal microflora) [42,43,44,45]. Several bioactive SFN analogues have been described [46]. In addition, an improvement of the stability of this compound was obtained after complexation with α-cyclodextrin (SFX-01) [47].

Interestingly, cruciferous vegetables have been reported to be beneficial for human health, as in the case of the prevention and therapy of cancer, including GBM [48,49,50]. The data available indicated that SFN may find a leading role in the development of therapeutic strategies for GBM [35,36,37].

The objective of the present study was to determine whether a combined treatment of SFN with molecules inhibiting miR-15b-5p might be proposed to increase SFN pro-apoptotic effects. In this respect, it should be underlined that a synergistic effect between SFN and a PNA against miR-15b-5p was found in the colon cancer cell line HT-29 [15]. The possible application of this strategy to GBM is of great interest, as it is a novel approach for GBM, and, even for the single treatments, the available data are scarce. In fact, no data on the effects of PNAs against miR-15b-5p are available on GBM model systems, and published studies on SFN are present, but none have focused on co-treatment with miRNA inhibitors.

## 2. Results

### 2.1. SFN-Mediated Induction of Apoptosis of Human Glioblastoma U251 Cells

**Figure 1:**
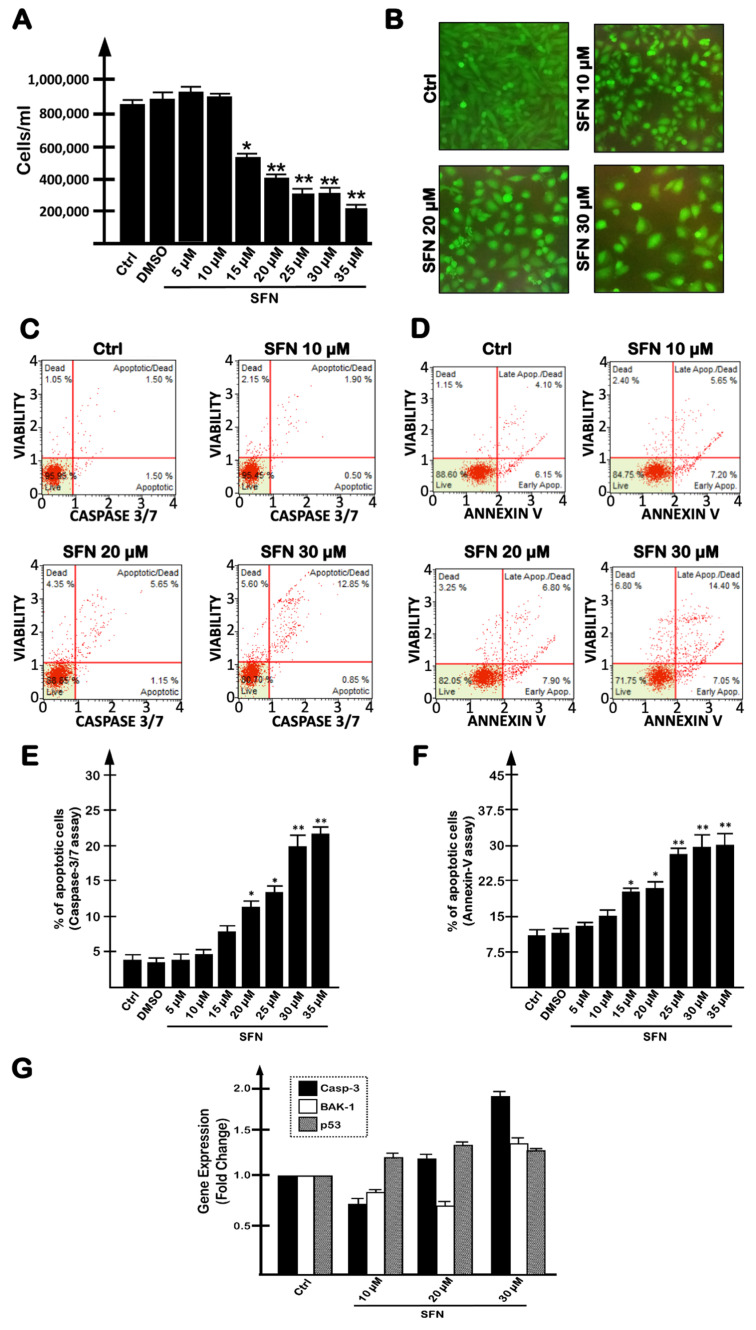
Effects of increasing concentrations of SFN on human U251 glioblastoma cells. Effects of SFN on cell growth (**A**), cell viability, evaluated by calcein AM staining (**B**), apoptosis (**C**–**F**) and apoptosis-related (Casp-3, BAK-1 and p53) mRNA production (**G**). Analyses were performed after 3 days of exposure to SFN. (**B**–**D**) Representative data. (**A**,**E**,**F**) Summary of three independent experiments. The data are presented as mean ± S.D. Statistical significance was verified using one-way ANOVA software (*, significant, *p* < 0.05; **, highly significant, *p* < 0.01).

In view of testing a combination of SFN and R8-PNA-a15b, we first analyzed the effect of an increasing concentration of SFN on U251 cells to establish the contribution of SFN alone to apoptosis in our cell model. When U251 cells were cultured in the presence of increasing concentrations of SFN (5–35 µM) for 3 days, cell growth was inhibited (Figure 1A,B) starting from a 15 µM concentration, and this effect was found to be associated with an increase in apoptosis (Figure 1C–F). Apoptosis was confirmed using the caspase-3/7 (Figure 1C,E) and the annexin-V (Figure 1D,F) assays. In Figure 1C,D, results from representative experiments are shown, whereas in Figure 1E,F, a summary of the data gathered from three independent experiments is presented. The vehicle for SFN (DMSO) exhibited no pro-apoptotic effects. The experiment shown in Figure 1G demonstrates that the pro-apoptotic effect of SFN is associated with an increase in caspase-3, BAK-1 and p53 mRNAs (see, in particular, the treatments with 30 µM SFN). Altogether, the results presented in Figure 1 demonstrate that SFN is a strong inhibitor of the cell growth of U251 cells (Figure 1A,B) and that this effect is associated with an induction of apoptosis (Figure 1C–F).

### 2.2. Targeting miR-15b-5p with the R8-PNA-a15b Molecule Down-Regulated miR-15b-5p and Induced Inhibition of U251 Cell Growth Associated with Pro-Apoptotic Effects

**Figure 2:**
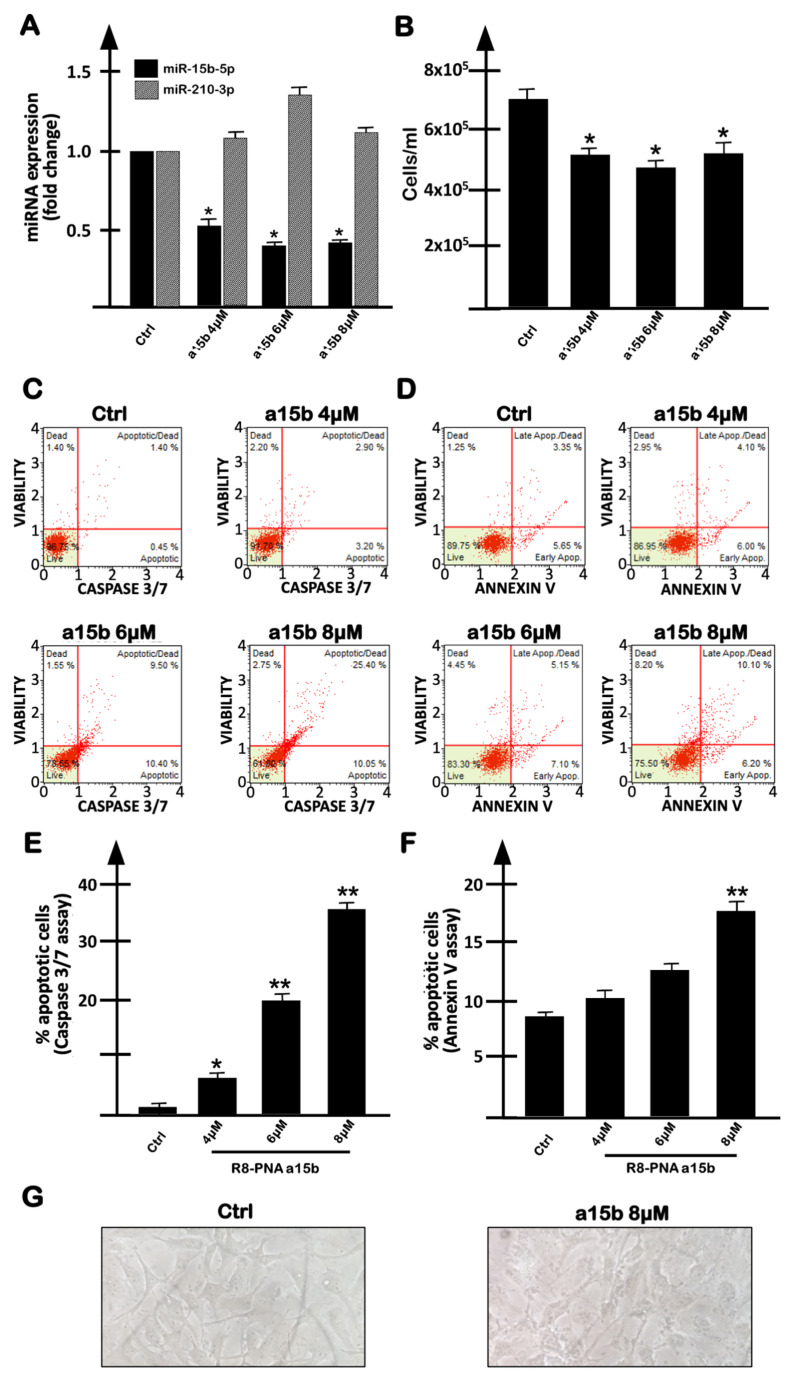
Effects on human U251 glioblastoma cells of increasing concentrations of R8-PNA-a15b-5p (a15b). (**A**) Effects of R8-PNA-a15b-5p on RT-qPCR amplification of miR-15b-5p (black boxes) and miR-210-3p (gray boxes). (**B**) Effects of R8-PNA-a15b on U251 cell growth. (**C**–**F**) Effects of R8-PNA-a15b on apoptosis. Analyses were performed after 3 days of exposure to R8-PNA-a15b. (**C**,**D**) Representative data of the effects on apoptosis: caspase-3/7 assay (**C**) and annexin-V assay (**D**). (**G**) Effects of R8-PNA-a15b on U251 morphology. (**A**,**B**,**E**,**F**) Summary of three independent experiments. The data are presented as mean ± S.D. Statistical significance was verified using one-way ANOVA software (*, significant, *p* < 0.05; **, highly significant, *p* < 0.01).

Considering the fact that the microRNA miR-15b-5p has been found to be up-regulated in aggressive GBM, and that its expression correlates with poor prognosis [30], we have designed a PNA (R8-PNA-a15b) able to interact with miR-15b-5p and expected to inhibit its biological functions. In order to maximize cellular uptake, this PNA molecule was functionalized with an octoarginine peptide (R8) [19,21]. The set of results obtained are reported in Figure 2 and show that the treatment of U251 cells with R8-PNA-a15b reduces miR-15b-5p-specific hybridization (Figure 2A), whereas it does not interfere with the expression of miR-210-3p, which is used as the internal control. The R8-PNA-a15b was found to inhibit, to some extent, cell growth (the effect was lower than SFN: compare Figure 1A and Figure 2B), and to induce apoptosis (Figure 2C–F). In Figure 2C,D, the results from representative experiments using caspase-3/7 (Figure 2C) and annexin-V (Figure 2D) assays are shown, whereas, in Figure 2E,F, a summary of the data gathered from three independent experiments is presented. Data indicate that the treatment with R8-PNA-a15b is able to induce apoptosis in a dose-dependent manner. No major alteration of U251 morphology was detectable, even when the highest concentrations of R8-PNA-a15b were employed (Figure 2G).

**Figure 3:**
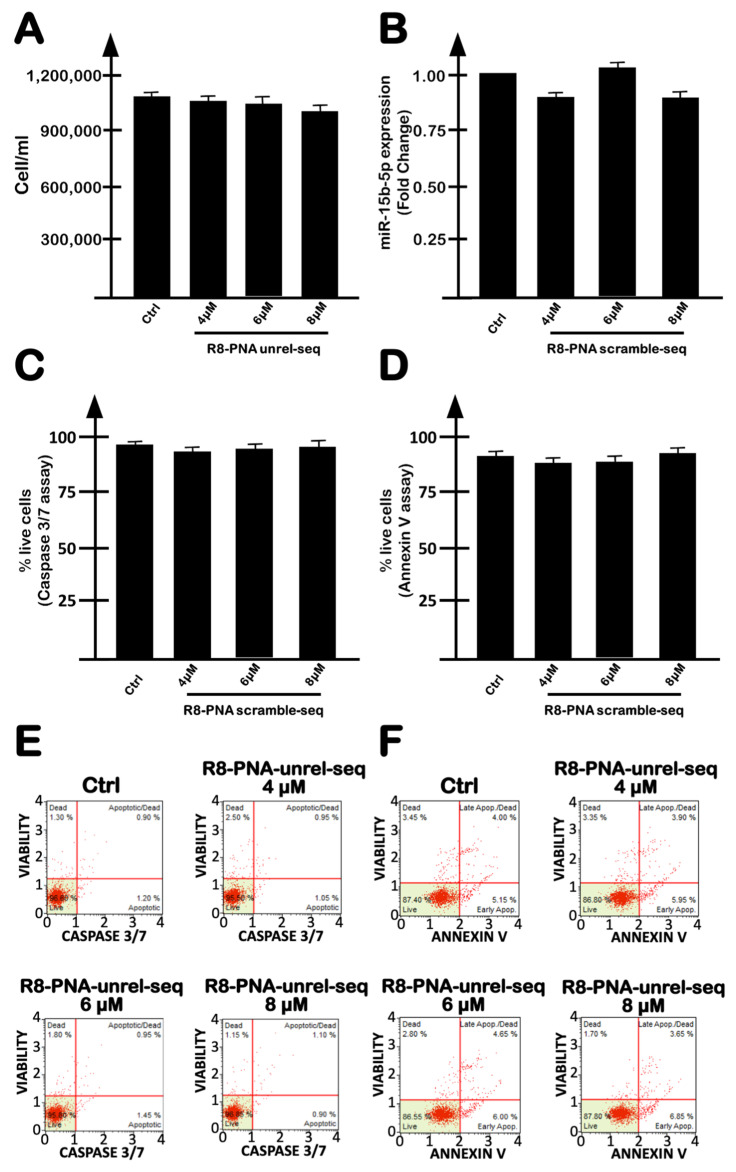
Effects on U251 cells of increasing concentrations of an unrelated sequence R8-PNA (R8-PNA-unrel-seq). Effects of this PNA on cell proliferation (**A**), on miR-15b-5p intracellular content (**B**) and on apoptosis induction (**C**–**F**). Apoptosis was evaluated using both caspase-3/7 (**C**–**E**) and annexin-V (**D**–**F**) assays. Analyses were performed after 3 days of exposure to the R8-PNA-unrel-seq. (**A**–**D**) Summary of three independent experiments. (**E**,**F**) Representative data. The data are presented as mean ± S.D. Statistical significance was verified using one-way ANOVA software.

With respect to miR-15b-5p-specific inhibition, inhibitory effects by R8-PNA-a15b were reproducibly observed on miR-15b-5p (Figure 2A, black boxes) but not on miR-210-3p (Figure 2A, gray boxes). In addition, the treatment of U251 cells with an unrelated sequence R8-PNA caused no biological effects (i.e., no inhibition of cell growth, no inhibition of miR-15b-5p and no induction of apoptosis) (Figure 3). These results support the conclusion that the effects of R8-PNA-a15b were specific.

### 2.3. Co-Treatment of U251 Cells with R8-PNA-a15b and Sulforaphane: Effects on Cell Growth and Apoptosis

**Figure 4:**
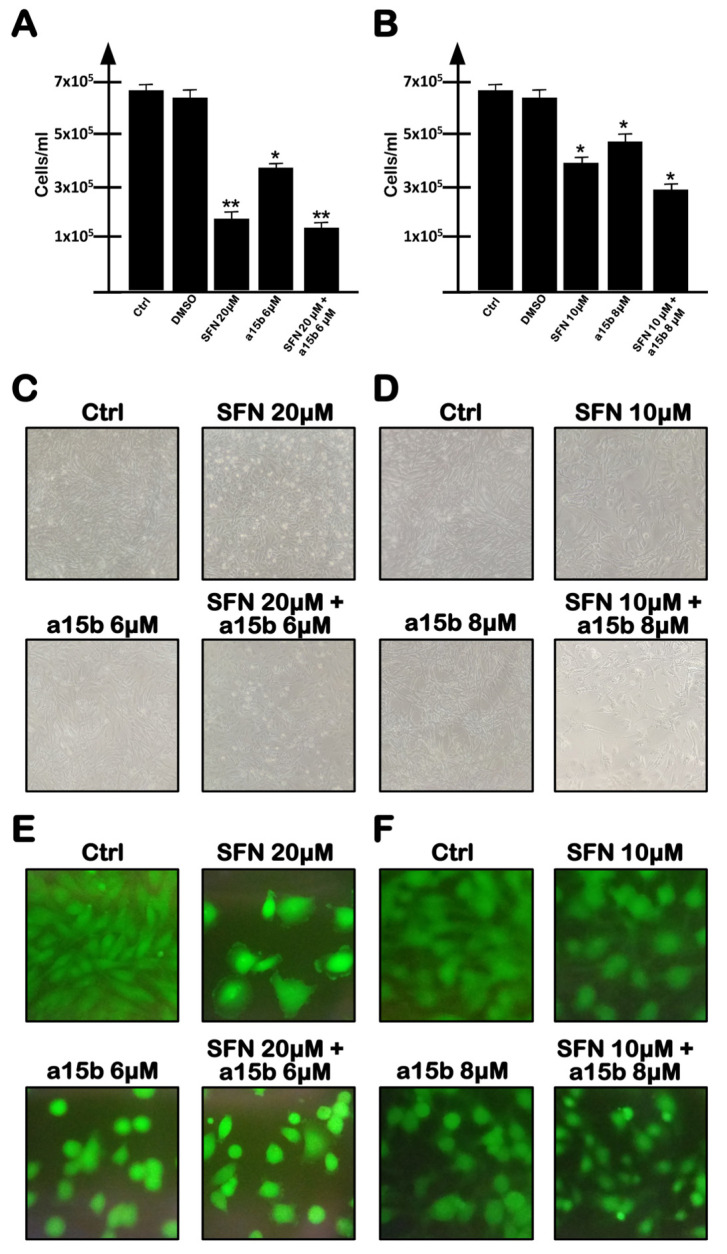
Effects of a combined treatment with R8-PNA-a15b and SFN. (**A**,**B**) Effects on in vitro proliferation of U251 cells. (**C**,**D**) Effects on cellular morphology: images at 10× magnification are reported. (**E**,**F**) Evaluation of cell viability: live cells are green stained using calcein-AM assay. U251 glioblastoma cells were untreated, or treated for 3 days with the SFN vehicle DMSO, or with indicated concentrations of SFN and/or R8-PNA-a15b. (**C**–**F**) Representative evaluations. (**A**,**B**) Summary of three independent experiments. The data are presented as mean ± S.D. Statistical significance was verified using one-way ANOVA software (*, significant, *p* < 0.05; **, highly significant, *p* < 0.01).

When the U251 cell line was cultured in the presence of singularly administered R8-PNA-a15b or SFN and the data obtained were compared with the U251 cell treated with a combination of R8-PNA-a15b and SFN, cell growth was only slightly inhibited (Figure 4). This was obtained using two combinations of SFN and R8-PNA-a15b: SFN 20 µM, R8-PNA-a15b 6 µM and SFN 10 µM, R8-PNA-a15b 8 µM.

In both cases, and in agreement with the results shown in Figure 1 and Figure 2, SFN was found to be more active in inhibiting cell growth. The combined treatment was found to only slightly increase the anti-proliferative effects of the treatment (a summary of three independent experiments is shown in Figure 4A,B, whereas representative microphotographs are shown in Figure 4C–F). The combination based on SFN 20 µM and R8-PNA-a15b 6 µM was proposed for the following experiments, considering that it leads to a more effective inhibition of U251 cell growth.

**Figure 5:**
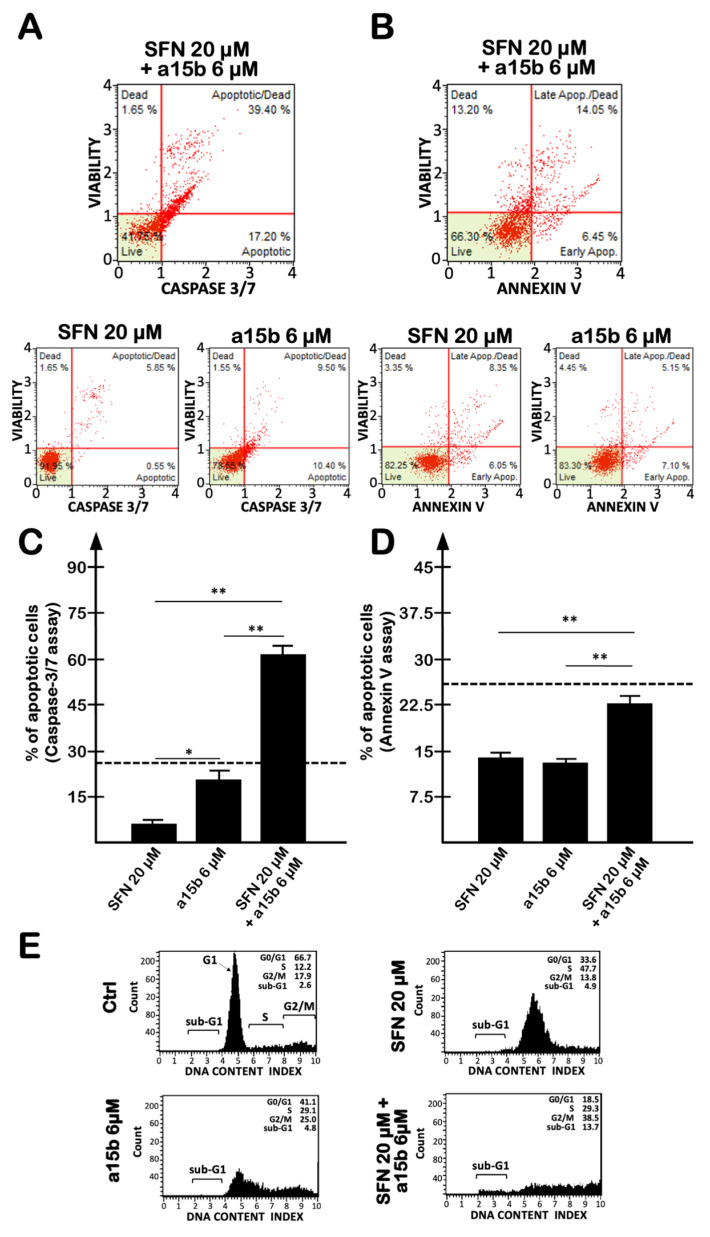
Effects of a combined treatment with the R8-PNA-a15b and SFN on apoptosis. Apoptosis was analyzed by caspase-3/7 (**A**,**C**) and annexin-V assays (**B**,**D**). U251 glioblastoma cells were untreated, or treated for 3 days with the SFN vehicle DMSO, with 20 µM of SFN, with 6 µM R8-PNA-a15b or both with 20 µM SFN and 6 µM R8-PNA-a15b. (**E**) Effects of single and combined treatments on cell cycle distribution. (**A**,**B**,**E**) Representative experiments. (**C**,**D**) Summary of three independent experiments. The data are presented as mean ± S.D. Statistical significance was verified using one-way ANOVA software (*, significant, *p* < 0.05; **, highly significant, *p* < 0.01). The dotted lines of panels (**C**,**D**) indicate the values of the sum of the single treatments.

In order to further verify whether SFN and R8-PNA-a15b synergistically induced the apoptosis of U251 cells, we employed intermediate concentrations of SFN (20 µM, see Figure 1) and R8-PNA-a15b (6 µM). The results obtained using the caspase-3/7 (Figure 5A,C) and annexin-V (Figure 5B,D) assays demonstrate that the induction of apoptosis in the combined treatment was significantly higher with respect to the single administration of SFN and R8-PNA-a15b.

Treatments were carried out for 3 days. Figure 5A,B show representative results obtained with caspase-3/7 (panel A) and annexin-V (panel B) assays, whereas Figure 5C,D reports a summary of the data obtained in three independent experiments. In this analysis, the background values found in the untreated control U251 cells have been subtracted. In particular, we found that the percentage of apoptotic cells in the combined treatment is higher than or closer to the sum of the single treatments, identified by the dotted line in panels C and D of Figure 5, respectively. It should be underlined that the most evident synergistic effects were found analyzing the % of total apoptotic cells when the caspase-3/7 assay was performed (Figure 5C) or when late apoptotic/dead cells were analyzed with both caspase-3/7 and annexin-V assays (see the representative results shown in Figure 5A,B). A possible explanation is that SFN efficiently activates caspase-3 [51].

In any case, the remarkable high induction of apoptosis in the combined treatment was fully confirmed by the analysis of the cell cycle, showing a presence of sub-G1 cells only in the combined treatment performed in the presence of both SFN and R8-PNA-a15b. In the experiment shown in Figure 5E, the sub-G1 fraction was 13.7% in the combined treatment, a value higher than 4.9% and 4.5%, exhibited by cell cultures treated with single administrations of SFN and R8-PNA-a15b, respectively. This result supports the hypothesis of synergistic effects, as the values of the % of the sub-G1 fraction is higher than the sum of the values of single treatments.

**Figure 6:**
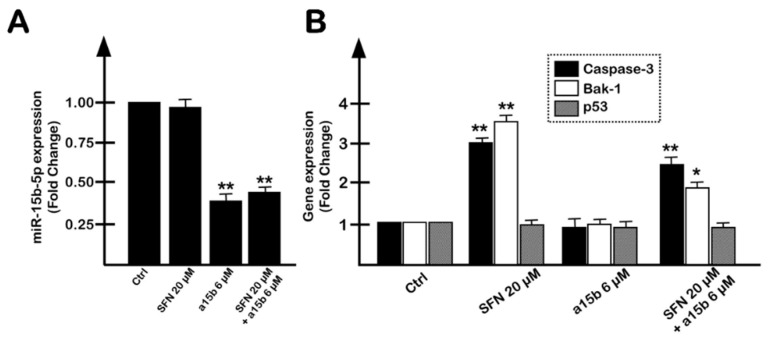
Effects of combined treatment of U251 cells with the R8-PNA-a15b and SFN on the expression of miR-15b-5p (**A**) and of caspase-3 (black bars), BAK-1 (white bars) and p53 (grey bars) mRNAs (**B**). The treatment was carried out for 3 days. The data are presented as mean ± S.D. Statistical significance was verified using one-way ANOVA software (*, significant, *p* < 0.05; **, highly significant, *p* < 0.01).

In order to obtain preliminary information possibly contributing to an explanation of the synergism between SFN and R8-PNA-a15b in inducing the apoptosis of U251 cells, experiments analyzing the levels of miR-15b-5p, and caspase-3, BAK-1 and p53 mRNAs demonstrated that the R8-PNA-a15b (and not SFN) is responsible for the miR-15b-5p down-regulation in treated U251 cells (Figure 6A) and that SFN (and not the R8-PNA-a15b) is responsible for the modulation of caspase-3 and BAK-1 mRNAs (Figure 6B). In these experimental conditions, p53 was not modulated.

### 2.4. The Effects on Apoptosis of the Co-Treatment of U251 Cells with R8-PNA-a15b and Sulforaphane Are Synergistic

**Figure 7:**
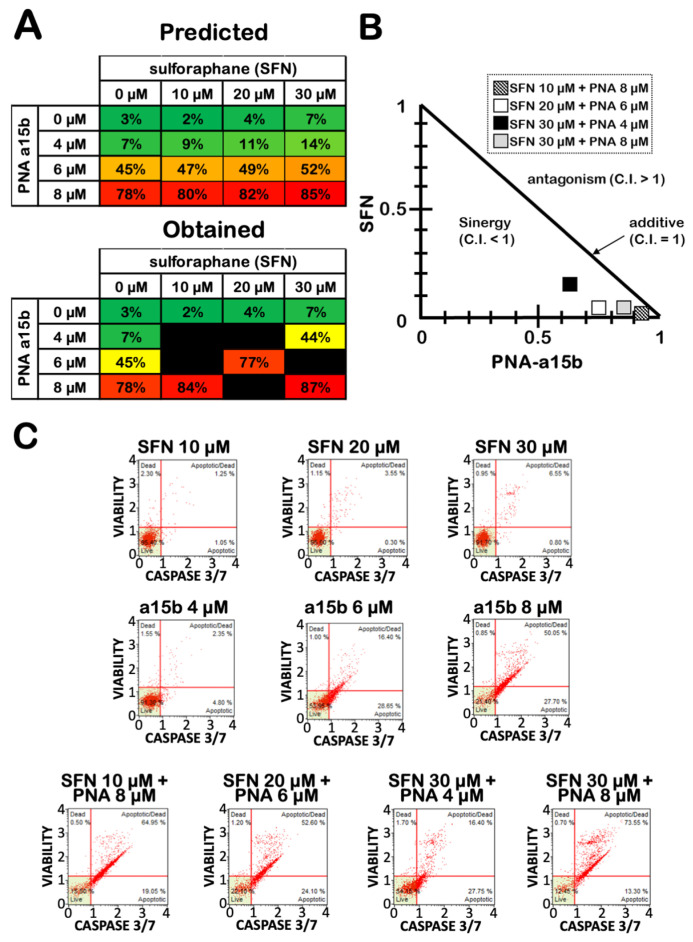
Synergic effects on apoptosis after combined treatments of U251 glioblastoma cells with SFN and R8-PNA-a15b. (**A**) Effects of increasing concentrations of SFN and R8-PNA-a15b on percentage of apoptotic cells. In the upper table of panel A, the expected proportion of apoptotic cells was predicted by calculating the sum of the values obtained using singularly administrated drugs. In the lower table of panel A, the obtained values during the co-treatment procedures are indicated. (**B**) Isobologram showing the combination index (C.I.) according to Chou and Talalay [53,54] method obtained by SFN and R8-PNA-a15b co-treatment. For C.I. calculation, compuSyn software was employed [51]. (**C**) Representative data of caspase-3/7 assay plots. All of the reported treatments were carried out for 3 days.

U251 cells were cultured for three days with 20 µM SFN in the absence or in the presence of R8-PNA-a15b, and the obtained results were compared to treatments performed using singularly administered SFN and R8-PNA-a15b. Apoptosis was detected using the caspase-3/7 assay. In Figure 7A, the predicted (i.e., the sum of the experimental values obtained after single administrations; upper part of the panel) and obtained (i.e., the experimental values obtained after combined treatments; lower part of the panel) are compared. Representative examples of the results are shown in Figure 7C. The results of the effects of combined treatments indicate that, in most of the drug combinations based on the use of the highest sub-optimal doses of SFN and R8-PNA-a15b, an induction of apoptosis was obtained with a very high efficiency. The obtained levels of apoptosis in combined treatments were higher than those predicted considering the sum of the apoptosis values obtained using singularly administered SFN and R8-PNA-a15b (compare in Figure 7A the tables concerning the predicted and observed values). Strong evidence of synergism (C.I. < 1) was obtained when the pharmacological additivity was determined by performing an isobologram analysis and calculating the combination index (C.I.) using the compuSyn software [52] according to the approach described by Chou and Talalay [53,54] (see Figure 7B). The most promising evidence of synergism was obtained with SFN 20 μM and R8-PNA-a15b 6 μM (C.I. = 0.789) and SFN 30 μM and R8-PNA-a15b 4 μM (C.I. = 0.790).

## 3. Discussion

Apoptosis has been proposed as a key target in the development of anti-tumor strategies [55,56,57,58,59,60], and the combination of several molecules involved in different pathways of apoptosis induction seems to be a promising strategy [61,62,63,64]. In this context, peptide nucleic acids (PNAs) are biomolecules of great interest [17,18] and were recently proposed in biomedical applications as anti-sense molecules targeting mRNAs, strand-invasion properties, decoy activity and potential gene editors [18,65]. PNAs have also been proposed as potent inhibitors of microRNA functions [19]. Since some microRNAs exert anti-apoptotic effects [66], PNAs interfering with anti-apoptotic miRNAs might be proposed for the development of anti-cancer treatments, such as PNAs targeting miR-221-3p, miR-155-5p and miR-15b-5p, which are strong inducers of the apoptosis of tumor cells [15,19,20,21,22].

On the other hand, apoptosis can be induced by a large variety of agents, and has been proposed as a key target in the development of anti-tumor strategies [55,56,57,58,59,60]. More recently, apoptosis was proposed to be induced by sulforaphane (SFN), one of the major biologically active products in broccoli (*Brassica oleracea* var. *Italica*) and other cruciferous vegetables [67,68,69,70,71,72,73]. In fact, sulforaphane was described as a strong inducer of apoptosis in several tumor systems, including hepatocellular carcinoma [67], prostate cancer [68], bladder cancer [69], colon cancer [70] and, relevant to the present study, GBM [71,72,73]. In this context, the data available indicated that SFN should be considered in the development of therapeutic strategies for GBM [35,36,37]. SFN was also employed in combined anti-cancer treatments [74,75].

The objective of the present study was to determine whether a combined treatment of SFN with molecules inhibiting miR-15b-5p might be proposed for increasing the SFN pro-apoptotic effects.

The results obtained clearly indicate that SFN and R8-PNA-15b synergistically act in inducing the apoptosis of the glioblastoma U251 cell line. In fact, the proportion of apoptotic cells in combined treatments is higher than the sum of the values obtained using singularly administered reagents (either SFN or the R8-PNA-a15b).

Our study is of interest when a comparison is made with several studies demonstrating the therapeutic potential of drug combination in glioblastoma. For example, De La Rosa et al. demonstrated a synergistic activity of temozolomide, 3-deazaneplanocin A and panobinostat on glioblastoma cell lines [76]. In another study, Sak et al. reported increased cytotoxic effects of combined treatments based on the use of alisertib, carboplatin and irinotecan [77]. Interestingly, the combined treatment should also be considered to overcome drug resistance, which is a major problem in the management of GBM [21,22]. Our protocol suggests that a combination of a natural product (sulforaphane) and a microRNA inhibitor (PNA-a15b) is another example of combined treatments for GBM. The data presented here demonstrate that this combined treatment leads to a very high proportion of apoptotic U251 cells (over 85%). Therefore, R8-PNA-a15b might be proposed in “combo-therapy” and associated with the use of other pro-apoptotic agents.

Our study has some limits. First of all, only one cell line was employed and no attempt has been conducted to determine the effects of the SFN/R8-PNA-15b combination on an in vivo experimental model system. Future experiments should be focused on other in vitro glioblastoma cell lines, in primary cells from GBM patients as well as on in vivo experimental model systems. Furthermore, the mechanism of action and target networks of both miR-15b-5p inhibitor PNAs and SFN should be studied in detail in order to identify novel molecular targets for therapeutic protocols.

In this respect, we also have to point out that the interplay between SFN and the R8-PNA-a15b was not clarified in our study. However, preliminary experiments that aimed to obtain information that possibly contributes to explaining the synergism between SFN and R8-PNA-a15b in inducing the apoptosis of U251 cells were performed. When the levels of miR-15b-5p, and caspase-3, BAK-1 and p53 mRNAs were analyzed, the results obtained indicate a differential activity of R8-PNA-a15b and SFN on the biomolecular markers, supporting the concept that the synergism on pro-apoptotic activity is likely to be associated to a differential effect of the single treatments on the overall gene expression. This study should, in the future, be expanded to other validated targets of miR-15b-5p and SFN. For instance, miR-15b-5p affects LATS2 [78], PTPN4/STAT3 [79], HPSE2 [80], axin2 [81] and RECK [32] in human cancers. On the other hand, SFN down-regulates CDK4/CDK6, inhibits tubulin polymerization [82], inhibits STAT3/HIF-1alpha/VEGF signaling [83], induces ERK1/2 and caspase-3 [51] and targets NRF2 [84] and other transcriptions factors [85].

The findings reported in this study are novel, as no report is available, to our knowledge, on a combination between SFN and miRNA-targeting molecules in GBM. In order to identify the molecular targets of this combined treatment in glioblastomas, further studies will be necessary, employing different in vitro established cell lines as well as primary cells from GBM patients. Finally, in vivo experiments will clarify whether this approach can be useful for the combo-therapy of GBM patients. While data on PNA-a15b are not available, the SFN concentrations used in our study are in line with those reported in studies in which in vitro and in vivo evaluations have been performed. For example, in the study by Bijangi-Vishehsaraei et al. [37] oral gavage delivery at 100 mg/kg of SFN significantly decreases the growth of ectopic GBM10 xenografts. In their study, the in vitro biological activity of SFN (i.e., reduction in survival and induction of apoptosis) was obtained using 10–50 μM concentrations.

Our study might encourage clinical trials focusing on the evaluation of the safety, tolerability and preliminary efficacy of peptide nucleic acid administered to patients, as in the case of NCT05212532 and NCT00127517, based on a PNA (EOM613/AVR 118) exhibiting immunomodulatory, antiviral and anticancer properties [86]. In this respect, several clinical studies based on SFN administration to cancer patients have been proposed, such as NCT03232138 (lung cancer), NCT01228084 (prostate cancer), NCT00982319 (breast cancer) and NCT01568996 (melanoma).

## 4. Materials and Methods

### 4.1. Reagents

All the chemicals and reagents were of analytical grade. For stock solutions of SFN (D,L-Sulforaphane, 574215-25MG, Merck Millipore, Burlington, MA, USA), 150 mM SFN in DMSO (D8418, Sigma-Aldrich, St. Louis, MI, USA) was prepared and stored at −20 °C (protected from light). The stock solutions of SFN were diluted 1:10 in DMSO just before use.

### 4.2. Synthesis of PNAs

The synthesis and chemical characterization of the R8-PNA-a15b (H-R8-TGT AAA CCA TGA TGT GCT-Gly-NH_2_) has been described elsewhere [15]. The R8-PNA-a15b was synthesized with an automatic synthesizer Syro I following a Fmoc protocol on a glycine pre-loaded resin (Fmoc-Gly-Rink amide ChemMatrix resin). After synthesis, PNA was precipitated in diethyl ether and purified in reverse phase HPLC as described [15]. The identity of the PNA was checked with UPLC-ESI system. The synthesis of PNA with an unrelated sequence (R8-PNA-unrel-seq, H-R8-TATCCAGTCAAGATCTAA-Gly-NH_2_) was similarly performed.

### 4.3. Cell Culture Conditions

U251 [87] cells were cultured in humidified atmosphere of 5% CO_2_/air in RPMI 1640 medium with L-Glutammine (EuroClone, Pero, Milano, Italy) supplemented with 10% fetal bovine serum (FBS, Biowest, Nuaillé, France), 100 units/mL penicillin and 100 µg/mL streptomycin (Pen-Strep, Sigma-Aldrich, St. Louis, MI, USA). U251 growth was monitored by determining the cell number/mL using a Z2 Coulter Counter (Coulter Electronics, Hialeah, FL, USA).

### 4.4. Cell Viability Determination

Cellular viability was analyzed by staining with calcein-AM (C1430; Thermo Fischer Scientific, Waltham, MA, USA) at final concentration of 500 nM. Cells were incubated for 30 min at 37 °C, washed twice with DPBS and visualized under a fluorescence microscope (Nikon Eclipse, Nikon Corporation, Minato, Tokyo, Japan). Viable cells were stained in green.

### 4.5. RNA Extraction

Cells were trypsinized (0.05% trypsin and 0.02% EDTA; Sigma-Aldrich, St. Louis, MI, USA), collected by centrifugation at 1200 rpm for 8 min at 4 °C, washed twice with DPBS 1X (Gibco, Thermo Fischer Scientific, Waltham, MA, USA) and lysed with Tri-Reagent (Sigma Aldrich, St. Louis, MI, USA), according to manufacturer’s instructions. The isolated RNA was washed once with cold 75% ethanol, dried and dissolved in nuclease free water. Obtained RNA was stored at −80 °C until use [15].

### 4.6. Quantitative Analyses of miRNAs

MicroRNA quantification was performed by real-time RT-qPCR and miRNA-specific primers and probes (Table 1) obtained from Applied Biosystems. Reverse transcriptase (RT) reactions were performed using TaqMan MicroRNA Reverse Transcription Kit (Applied Biosystems, Thermo Fischer Scientific, Waltham, MA, USA) according to the manufacturer’s protocol. All RT reactions, no-template controls and RT-minus controls were run in duplicate using TaqMan Universal PCR Master Mix, no AmpErase UNG 2X (Applied Biosystems, Thermo Fischer Scientific, Waltham, MA, USA) and the CFX96 Touch Real-Time PCR Detection System (BioRad, Hercules, CA, USA), as described elsewhere [15]. The Bio-Rad CFX Manager Software (Bio-Rad, Hercules, CA, USA) was used to collect and analyze data. The relative expression was calculated using the comparative cycle threshold method, and snRNA U6 and hsa-let-7c-5p were used as reference sequences, as previously reported [15,19,20].

### 4.7. Analysis of Apoptosis-Related Genes by RT-qPCR

The expression of genes involved in apoptotic pathway was verified by RT-qPCR, as described elsewhere [19,20,21,22]. RNA was reverse-transcribed using random hexamers and TaqMan Reverse Transcription PCR Kit (Thermo Fischer Scientific, Waltham, MA, USA). The quantitative real-time PCR (qPCR) assays were performed using gene-specific fluorescently labeled probes. The assays used that were specific for caspase-3 (assay ID: Hs.PT56a.24277143), p53 (assay ID: Hs.PT.58.123122) and BAK1 (assay ID: Hs.PT.56a.40435467) [88,89,90] were purchased from IDT (Integrated DNA Technologies, Coralville, IA, USA). The relative mRNA content was calculated using the comparative cycle threshold method, and fold change was calculated as 2^−ΔΔCT^. The internal reference sequence was the human RPL13A (Thermo Fischer Scientific, Waltham, MA, USA, assay ID: Hs04194366_g1). All RT-qPCR reactions were performed in duplicate, as reported elsewhere [15,17].

### 4.8. Analysis of Apoptosis

Apoptosis assays were performed as described elsewhere using the Muse Cell Analyzer instrument (Millipore Corporation, Billerica, MA, USA). The Muse Annexin V & Dead Cell Kit and the Muse Caspase-3/7 Kit were employed [15,17]. These assays differentiate viable non-apoptotic cells from early apoptotic, late apoptotic and dead cells.

### 4.9. Analysis of the Combined Treatment with SFN and R8-PNA-a15b

The analysis of the combined treatments was carried out using the method developed by Chou and Talalay [53,54]. The combination index (C.I.) was calculated using the CompuSyn tool for drug synergy analysis (freely available). The Compusyn software defines the effects as synergic when the interactions between the drugs exhibit a C.I. value less than 1; C.I. values lower than 0.5 indicate strong synergic effects of the combined treatments. By contrast, C.I. values close to 1 indicate additive effects and values higher than 1 indicate antagonistic effects [52].

### 4.10. Statistics

All the data were normally distributed and presented as mean ± S.D. Statistical differences between groups were compared using one-way ANOVA (analyses of variance between groups) software. Statistical differences were considered significant when *p* < 0.05 (*), and highly significant when *p* < 0.01 (**).

## 5. Conclusions

The data presented in the present study strongly support the concept that the combined treatment of tumor cells with PNA targeting microRNAs (in this study, a PNA targeting miR-15b-5p) and anti-tumor agents (in this study, sulforaphane) is a promising approach to develop experimental protocols that are finalized to increase the efficacy of the treatments and to limit, at least in theory, the side effects of the employed drugs. However, before proposing this approach for clinical trials, some issues should be analyzed: (a) preclinical studies using GBM animal models to test the efficacy of the combined therapy, including the use of suitable delivery strategies; (b) the concentrations of SFN and R8-PNA-a15b in combined treatments, to be further analyzed to verify whether a sub-μM range could still be effective; (c) omics analyses, to identify concentrations causing minimal alterations of transcriptomics and proteomics in treated GBM cells.

## Figures and Tables

**Table 1:** List of assays employed for miRNA detection.

Target Name	Assay ID
hsa-miR-15b-5p	000390
hsa-miR-210-3p	000524
hsa-snRNA U6	001973
hsa-let7c-5p	000379

## Data Availability

All the data will be available upon request to the corresponding authors.

## Sample Availability

Samples of the compounds will be available unless out of stock.
